# Free water, occipital volume and changes in depressive symptoms in the *LifeAfter90* study

**DOI:** 10.1038/s41598-026-54542-9

**Published:** 2026-05-23

**Authors:** Alexander Ivan B. Posis, Ruijia Chen, Hilary L. Colbeth, Molly R. LaPoint, Kristen M. George, Paola Gilsanz, M. Maria Glymour, Pauline Maillard, Yonas Tesfagabr Abraham, María M. Corrada, Rachel A. Whitmer

**Affiliations:** 1 Department of Public Health Sciences, University of California, Davis, Medical Sciences 1-C, One Shield’s Ave, Davis, CA 95616 USA; 2 Department of Epidemiology, Boston University School of Public Health, Boston, MA USA; 3 Kaiser Permanente Northern California Division of Research, Pleasanton, CA USA; 4 Department of Neurology, University of California, Davis, Davis, CA USA; 5 Department of Neurology, University of California, Irvine, Irvine, CA USA; 6 Department of Epidemiology and Biostatistics, University of California, Irvine, Irvine, CA USA

**Keywords:** Depression, Neuroimaging, Older adults, Oldest-old, Prospective cohort, Neurology, Epidemiology, Depression

## Abstract

Though depression impacts over 10% of the oldest-old, the contribution of brain health on changes in depression is unknown. This study included 225 participants from *LifeAfter90* with magnetic resonance imaging, amyloid positron emission tomography (PET), and repeated Geriatric Depression Scale (GDS) data. We fit linear mixed-effects models for associations of neuroimaging markers with depressive symptom change adjusted for age, gender, race and ethnicity, and education. At baseline, average GDS scores were 2.6 (SD = 2.3) out of 15 possible points, average age was 93.3 (SD = 2.3) years, 56% female, and 22% Black, 25% Asian, 18% Hispanic/Latino, 28% Non-Hispanic White, and 7% as Multiracial/other. Greater baseline occipital volume was associated with slower GDS increase (β = − 0.29; 95% CI − 0.54, − 0.05). Greater baseline free water was associated with faster GDS increase (β = 0.31; 95% CI 0.05, 0.57). Estimates for other neuroimaging biomarkers leaned towards slower, but non-significant, increases in GDS. Estimates were not in the expected direction for amyloid PET and null for frontal cortex volume. Our findings suggest that lower cortical volume and greater white matter injury is associated with increases in depressive symptoms among the oldest old, even for those with low levels of baseline depressive symptoms.

**Supplementary Information:**

The online version contains supplementary material available at 10.1038/s41598-026-54542-9.

## Introduction

Over 10% of adults over age 90 in the United States have depression^1^, a leading cause of disability-adjusted life years^2^. Understanding the determinants of depression among the oldest old is a high priority because the global population over age 90 is projected to increase from 25 million in 2024 to over 147 million by 2060^3^. Moreover, the presentation and management of depression is complex given multiple comorbidities at older age^4^, justifying the need to etiologically clarify potential links between brain health and depressive symptom change. Given the expected growth of the oldest old population and negative impact of depressive symptoms, a clearer understanding of associations of brain health with trajectories of depressive symptoms among populations over age 90 is warranted.

Depressive symptoms are associated with subtle brain changes that suggest poor brain health. For example, increasing trajectories of depressive symptoms in earlier-life are associated with a greater risk of dementia^5^. Depressive symptoms are also associated with indicators of poor white matter integrity, such as greater presence of white matter hyperintensities (WMH)^6–9^. Past research among younger-old populations suggests that lower hippocampal volumes and fractional anisotropy (FA) and increased amyloid accumulation are associated with subsequent increases in depressive symptoms^10–12^. However, research on the reverse effects, how brain health impacts subsequent depressive symptom trajectories, is limited. The biological mechanism between brain health and depressive symptoms may occur through several mechanisms such as vascular risk factors. For example, arterial stiffening is associated with less cortical volume and white matter integrity^13,14^, which can contribute to the development of depressive symptoms^15^. Poor brain health may also result in reduced social contact^16^, followed by increases in depressive symptoms.

There is limited understanding of the contributions of brain health biomarkers after age 90 on changes in depressive symptoms. Prior research largely focuses on whether depressive symptoms precede poorer brain health^5–9^. Information on whether certain cortical and white matter-related features impact changes in depressive symptoms would improve our understanding of the dynamic nature of depressive symptoms among those with exceptional longevity. Moreover, later-life subsyndromal depression is associated with poor negative health outcomes^17,18^; this highlights the importance of identifying depressive symptoms even at lower levels that are perhaps not sufficient for a formal depression diagnosis^19^. Given objective measures of brain health, findings may provide stronger evidence of the negative depressive symptom consequences of poor brain health. Therefore, the objective of this study was to investigate associations between several neuroimaging-based brain biomarkers with trajectories of depressive symptoms among racially and ethnically diverse adults aged 90 to 103 years. We hypothesized that lower cortical volume, less white matter integrity, and greater amyloid at baseline would be associated with faster increases in depressive symptoms.

## Methods

We used data from the *LifeAfter90* study, an ongoing longitudinal cohort study of lifecourse determinants of cognitive and brain aging among the oldest old. The *LifeAfter90* study began in July 2018 with study visits occurring every 6 months. Eligible participants were aged 90 years and older at enrollment, were long-term members of Kaiser Permanente Northern California (KPNC), and resided in the San Francisco Bay and Sacramento areas of California. Inclusion criteria included ability to provide informed consent, no diagnosis of dementia or other neurodegenerative disease, not in hospice care, and no dialysis in their electronic medical record at the time of enrollment. All *LifeAfter90* participants were asked if they were interested in completing a neuroimaging exam. A random sample of approximately 25% of participants who indicated interest were selected for structural magnetic resonance imaging (MRI) and amyloid positron emission tomography (PET). Study protocols were approved by the KPNC and University of California, Davis Institutional Review Boards (#1279409). All participants provided informed consent. Research was performed in accordance with the Declaration of Helsinki. The present study followed the Strengthening the Reporting of Observational Studies in Epidemiology (STROBE) reporting guidelines for cohort studies.

Of the 999 *LifeAfter90* study participants recruited as of December 2022, 226 had imaging data. Of these, we excluded 1 participant without depressive symptom data at baseline, yielding a final analytic sample of 225 participants. At the first opportunity, image acquisition occurred once per participant over a maximum follow-up of 3.87 years and 8 possible visits. Baseline depressive symptoms and demographic data were obtained from the visit closest to imaging, which could occur before or after imaging, and then followed forward. For example, if a participant had 5 GDS visits and imaging was acquired on visit 3, we considered GDS data from visit 3 and onward. The full study period was July 2018 to December 2022.

### Measures

MRI acquisition and processing were performed using standardized protocols. Briefly, MRIs were acquired using 3T Siemens TrioTrim or Prisma Fit scanners at a single timepoint. Neuroimaging-based measures of total intracranial volume (ICV), total and regional gray matter volume, including log-transformed WMH volume, as well as mean free water (FW) and fractional anisotropy (FA) were derived from validated processes^20–27^. Full details on MRI acquisition and processing are found in the Supplementary Methods. Neurodegeneration markers included volumetric measures (cm^3^) of total cerebrum gray matter, total and bilateral hippocampus, regional cortical volume, and cerebrospinal fluid (CSF) lateral and third ventricular volume. To assess amyloid accumulation, PET scans used amyloid-binding fluorine-18 radiotracer florebatapir (Avid Radiopharmaceuticals) and were processed using Alzheimer’s Disease Neuroimaging Initiative protocols (see https://adni.loni.usc.edu/methods/pet-analysis-method/pet-analysis/ and Supplementary Methods). Mean standardized uptake value ratio (SUVR) was calculated to quantify regional tracer uptake across voxels relative to the whole cerebellar gray matter reference region^27^. Amyloid PET was quantified as mean SUVR across frontal, occipital, parietal, and temporal regions. Regional MRI and WMH volumes were ICV-corrected by regressing each volume onto ICV and calculating residual values. All baseline brain biomarker measures were Z-scored for comparability.

Depressive symptoms were measured using the 15-item Geriatric Depression Scale (GDS)^28,29^. At each visit, participants were asked 15 ‘yes’ or ‘no’ questions. Full details on each question are shown in Supplementary Table 1. After reverse scoring items 1, 5, 7, 11, and 13, responses to all items were summed to generate a summary score (possible range = 0–15) with 0 indicating no depressive symptoms and 15 indicating all measured symptoms. Greater GDS suggests increasing depressive symptoms and scores ≥ 4 are suggestive of clinical depression in study populations aged 90+ years^30,31^.

Covariates considered to be potential confounders included age at neuroimaging scan, gender (men; women), race and ethnicity (African American/Black; Asian; Hispanic/Latino; Multiracial/Other; White), and highest reported level of education (≤ High School/GED; Some College or Tech/Trade School; College; Graduate School). Time-varying verbal episodic memory and executive function, from the same visit as the GDS, were derived from the Spanish and English Neuropsychological Assessment Scale (SENAS)^32,33^. The verbal episodic memory composite score was derived from a word list learning test^32^. The executive function composite score was derived from tasks pertaining to category fluency, phonemic fluency, and digit-span backward and list sorting tasks for working memory^33^. SENAS scores were z-standardized using the study population’s baseline for each cognitive domain score.

### Statistical analysis

We described participant characteristics using mean (standard deviation [SD]) or median (minimum, maximum) for continuous variables, and counts (percentages) for categorical variables. To test associations of baseline brain biomarkers with longitudinal change in depressive symptoms (i.e., trajectories), we fit linear multivariable mixed-effects models with random intercepts and slopes, with separate models for each baseline brain biomarker. We used years since baseline as the timescale to assess within-participant change. Models included the main effects of the brain marker, time, and a brain marker-by-time interaction term. To assess the impact of bias due to selection into the *LifeAfter90* imaging subcohort, we repeated our primary analyses with inverse probability of selection weights (IPSWs)^34^. IPSWs were calculated for each participant by computing the inverse of propensity scores for selection into the imaging cohort, conditional on baseline GDS, age, age-squared, SENAS executive function and verbal episodic memory, SENAS-by-age interactions, gender, race and ethnicity, and education. IPSWs were then stabilized by the probability of selection into the imaging subcohort conditional on baseline GDS scores, and then trimmed at the 5th and 95th percentiles. IPSWs created a pseudopopulation of equivalent size to the observed after selection where participants in the present analysis are upweighted to account for participants who were not included in the *LifeAfter90* imaging subcohort, conditional on propensity scores. For visualization, we plotted estimated trajectories of GDS over time by z-scored values of neuroimaging markers. All models adjusted for baseline age, gender, sex, race and ethnicity, and education.

Three sensitivity analyses were performed. First, because depression is a noted risk factor for dementia^35^, we repeated our primary analysis with additional adjustment for time-varying SENAS verbal episodic memory and executive function scores. Second, we assessed the impact of reverse causality by repeating our main analysis after excluding *n* = 60 with GDS ≥ 4 and *n* = 39 with GDS ≥ 5 at baseline who may have clinically significant depressive symptoms^30,31^. Third, we explored potential moderation of associations by gender and race and ethnicity by comparing models with and without a brain biomarker-by-modifier-by-time interaction using likelihood ratio tests.

All statistical analyses were conducted using R version 4.4.0 (R Core Team 2024).

## Results

### Study population characteristics

The average (SD) age of the 225 participants at imaging was 93.3 (2.3) years (median [range] = 92.7 [90.5, 103]; Table 1). Most participants were women (56%) and had at least some college or tech/trade school education (78%). The cohort was diverse with respect to race and ethnicity: 22% identified as African American/Black, 25% as Asian, 18% as Hispanic/Latino, 28% as White and 7% as multiracial or another racial and ethnic group. At baseline, mean (SD) was GDS scores were 2.6 (2.3) out of a possible 15 points. GDS scores were elevated (≥ 4, suggesting clinically significant depressive symptoms) for 27% of participants^24,25^. Compared with the full *LifeAfter90* cohort, the imaging subcohort had a higher proportion of men (44% vs. 39%), participants who identified as White (28% vs. 26%), and participants with at least some college or tech/trade school education (78% vs. 71%).

## Associations of baseline brain biomarkers with longitudinal change in depressive symptoms

**Fig. 1:**
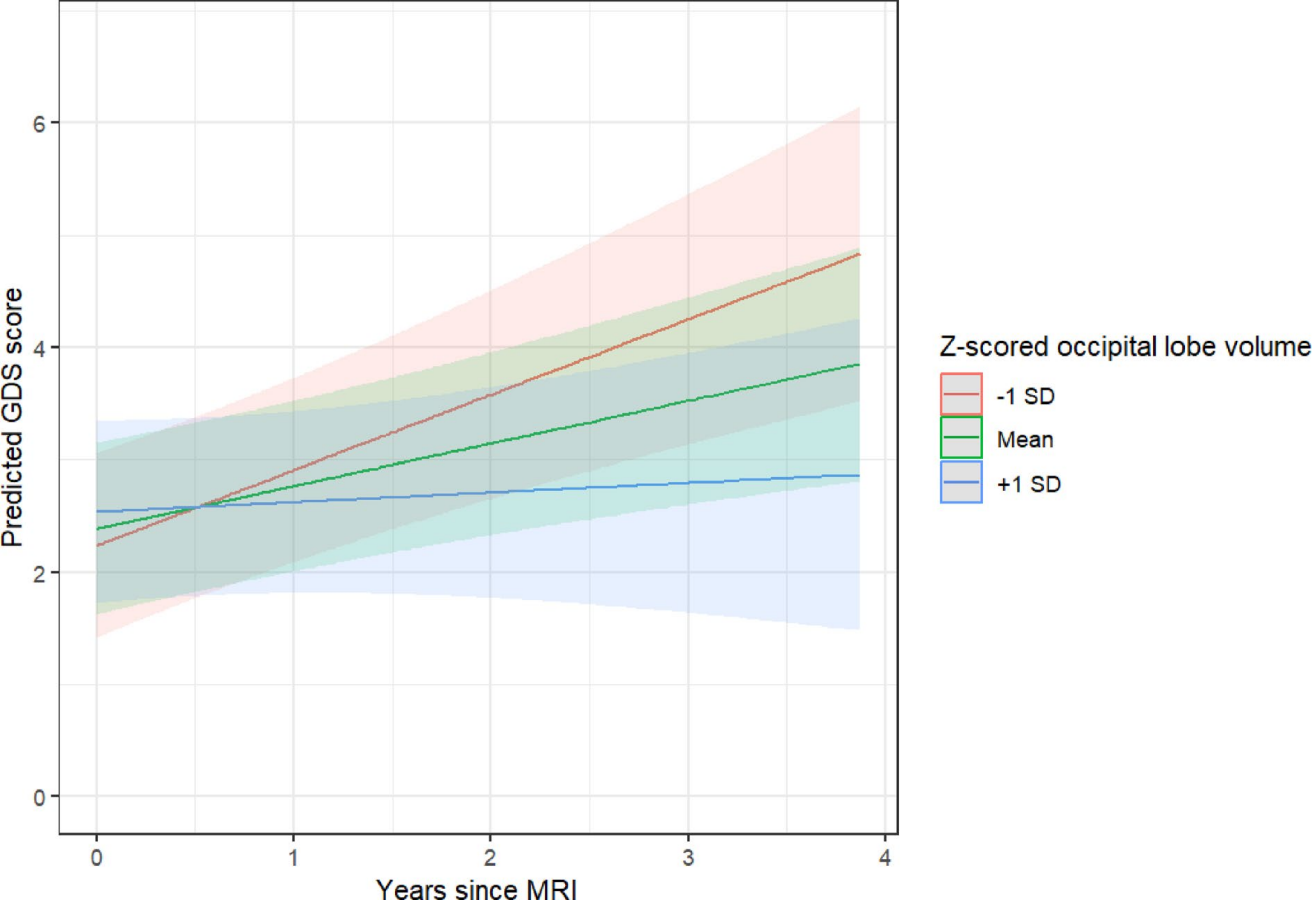
Predicted Geriatric Depression Scale (GDS) scores over time, where higher scores suggest greater depressive symptom severity, stratified by z-scored occipital lobe volume, adjusted for age, gender, race and ethnicity, and education.

**Fig. 2:**
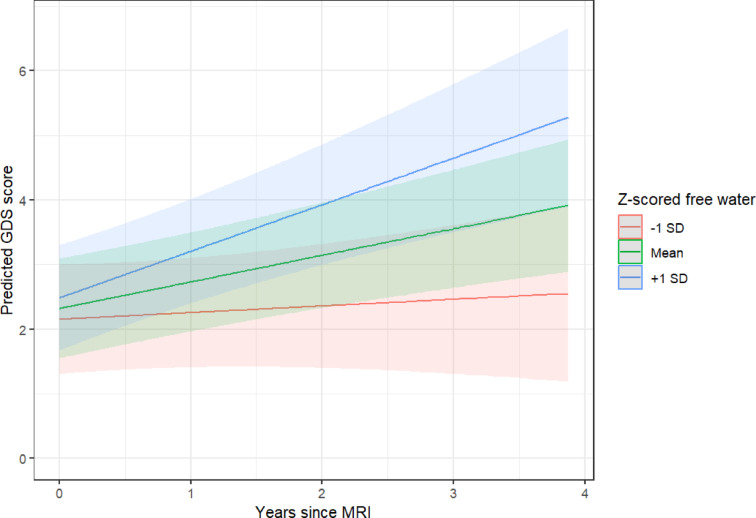
Predicted Geriatric Depression Scale (GDS) scores, where greater scores suggest greater depressive symptom severity, by z-scored free water and over time adjusted for age, gender, race and ethnicity, and education.

Over 3.87 years of follow-up (mean = 0.96; SD = 1.15), GDS gradually increased at an average annual rate of 0.35 (95% CI 0.13, 0.58) points over the possible 7 visits (Supplementary Fig. 1). In unweighted models, greater baseline occipital cortex volume was associated with slower increase in GDS scores (β_occipital volume by time_ = − 0.29; 95% CI − 0.54, − 0.05; Table 2; Fig. 1). Meaning, a single standard deviation increase in baseline occipital cortex volume at baseline was associated with a 0.29-point slower annual increase in GDS scores. Greater FW fraction was associated with a faster increase in GDS scores (β_FW by time_ = 0.31; 95% CI 0.05, 0.57; Table 2; Fig. 2). There were no differences in GDS change over time in relation to the other baseline brain biomarkers. However, estimates were generally in the expected direction such that greater baseline CSF, cortex, cerebrum and hippocampal gray matter volumes (see Supplementary Fig. 2 for an example of predicted GDS scores by hippocampal volume), and indicators of better white matter integrity leaned towards slower, but non-significant, increases in GDS. Estimates were not in the expected direction for amyloid SUVR (β_SUVR by time_ = − 0.08; 95% CI − 0.29, 0.12) and null for frontal cortex volume (β_frontal cortex by time_ = < 0.01; 95% CI − 0.21, 0.22). Results were similar after applying IPSWs to account for potential selection bias from the overall *LifeAfter90* cohort into the imaging subcohort.

**Table 1:** Participant characteristics by cohort.

Characteristic		Present study with neuroimaging (*n* = 225)	Full LifeAfter90 cohort at baseline (*n* = 999)
Age at baseline	Median [Min, Max]	–	91.6 [90.1, 105]
Age at imaging	Median [Min, Max]	92.7 [90.5, 103]	–
Gender, n (%)	Men	99 (44.0%)	386 (38.6%)
	Women	126 (56.0%)	613 (61.4%)
Race/ethnicity, n (%)	African American/Black	49 (21.8%)	228 (22.8%)
	Asian	56 (24.9%)	236 (23.6%)
	Hispanic/Latino	41 (18.2%)	189 (18.9%)
	Multiracial/Other	16 (7.1%)	85 (8.5%)
	White	63 (28.0%)	261 (26.1%)
Education, n (%)	≤High School/GED	49 (21.8%)	289 (28.9%)
	Some College or Tech/Trade School	73 (32.4%)	352 (35.2%)
	College	55 (24.4%)	166 (16.6%)
	Graduate School	48 (21.3%)	188 (18.8%)
Baseline GDS	Mean (SD)	2.63 (2.30)	2.90 (2.64)
Baseline GDS ≥4	n (%)	60 (26.7%)	282 (28.2%)

Race/ethnicity category “other” includes other, multiple, refused, and missing; *n* = 52 missing GDS in full *LifeAfter90* cohort.

**Table 2:** Unweighted and inverse probability of selection (IPSW) weighted longitudinal association of 1 SD increase in brain biomarkers with Geriatric Depression Scale scores over time.

Brain biomarker by time	Unweighted estimates	IPSW estimates
	β (95% CI)	β (95% CI)
Amyloid		
SUVR	− 0.08 (− 0.29, 0.12)	− 0.07 (− 0.27, 0.14)
Cerebrospinal fluid volume		
Lateral ventricle	0.17 (− 0.06, 0.41)	0.17 (− 0.11, 0.44)
Third ventricle	0.11 (− 0.12, 0.34)	0.14 (− 0.12, 0.40)
Cortex volume		
Frontal	< 0.01 (− 0.21, 0.22)	− 0.03 (− 0.29, 0.22)
Occipital	− 0.29 (− 0.54, − 0.05)	− 0.29 (− 0.58, − 0.01)
Parietal	− 0.22 (− 0.46, 0.02)	− 0.17 (− 0.45, 0.12)
Temporal	− 0.18 (− 0.41, 0.05)	− 0.13 (− 0.40, 0.13)
Gray matter volume		
Total cerebrum gray	− 0.18 (− 0.41, 0.04)	− 0.18 (− 0.43, 0.08)
Total hippocampus	− 0.16 (− 0.38, 0.06)	− 0.18 (− 0.43, 0.07)
Left hippocampus	− 0.13 (− 0.34, 0.08)	− 0.15 (− 0.40, 0.09)
Right hippocampus	− 0.16 (− 0.38, 0.07)	− 0.16 (− 0.42, 0.10)
White matter integrity		
Free water	0.31 (0.05, 0.57)	0.30 (0.00, 0.60)
Fractional anisotropy	− 0.30 (− 0.64, 0.03)	− 0.31 (− 0.69, 0.07)
Log of WMH	0.15 (− 0.12, 0.42)	0.19 (− 0.12, 0.49)

All brain biomarkers were z-standardized and were residual values that accounted for intracranial volume.

GDS is scored such that higher score suggests greater depressive symptom severity (range 0–15).

IPSW estimates account for selection into the LifeAfter90 imaging subcohort.

Models adjusted for age, gender, race/ethnicity, and education.

Abbreviations: SUVR = standardized uptake value ratio, WMH = white matter hyperintensities.

### Sensitivity analyses

In our first sensitivity analysis which adjusted for time-varying verbal episodic memory and executive function in the same model, we observed beta coefficients of similar magnitude across all models, but only remained significant for FW (β_FW by time_ = 0.28; 95% CI 0.03, 0.54; Supplementary Table 2). In the next sensitivity analysis to assess reverse causation, beta coefficients and directions of associations across all models were similar after excluding participants with GDS scores of ≥ 4 and ≥ 5, but were attenuated for occipital volume after excluding GDS ≥ 5 (β_occipital volume by time_ = − 0.25; 95% CI − 0.51, 0.01; Supplementary Table 3). In the last sensitivity analysis, we did not detect moderation in associations by gender or by race and ethnicity (all p-values for interaction > 0.05; Supplementary Table 4).

## Discussion

In this prospective cohort study of 225 racially and ethnically diverse adults aged 90 to 103 years, greater baseline occipital volume was associated with a slower increase in GDS scores during up to 3.87 years of follow-up. We also found that greater baseline FW was associated with faster increases in GDS scores. These results were robust to IPSWs which accounted for selection into the *LifeAfter90* imaging cohort. Our results were attenuated after adjustment for time-varying executive function and verbal episodic memory.

Depressive symptoms, measured by the GDS, in this cohort increased at an average annual rate of 0.35 (95% CI 0.13, 0.58). This estimate is marginal in comparison to a recent meta-analysis which suggests that 3.81 (95% CI 3.59, 4.04) is a minimally detectable change^36^. Generalizability of this minimally detectable change estimate to our cohort may be difficult given demographic differences, such as older age and racial and ethnic distributions. Despite low baseline levels and small change in GDS, we found that total free water and occipital volumes were associated with change in GDS. When considering the potential downstream impacts of depressive symptoms, even with modest changes, our findings are notable given associations of subsyndromal depression with greater hazard of mortality and rate of morbidity^17–19^. Moreover, depressive symptoms can impact social connection and loneliness, which may increase one’s risk of poor health outcomes such as cardiovascular disease and dementia^35,37^.

We found that increased FW within the overall white matter was associated with a faster increase in GDS scores, but changes in GDS were not associated with our other indicators of white matter integrity which included FA and WMH. Interestingly, a recent study among 381 participants aged 72 to 92 years with 2 years of follow-up evidenced that greater GDS was associated with reduced FA, particularly in the superior frontal gyrus, corpus callosum, right posterior thalamic radiation, and the left superior longitudinal fasciculus^10^. Although the previous study of 381 spanned a wider age range and only measured GDS at two timepoints^10^, which may not accurately capture the trajectory of all depressive symptoms, it emphasizes the need for further investigation into the potential role of FW within specific white matter microstructural tracts on the course of depressive symptoms.

Our white matter integrity findings potentially correspond with the vascular depression hypothesis which posits that cerebrovascular disease via disconnection, inflammatory, and hyperfusion mechanisms play a role in the development of depressive symptoms^15^. In the inflammatory hypothesis, for example, stress can lead to inflammatory responses that may coincide with neurodegeneration and potentially increase the presence of depressive symptoms^38^. In our study, we found a significant association between greater FW and faster rate of GDS increase. This is plausible given that greater FW is associated with arterial stiffening^14,39^, which in turn could impact GDS score trajectories through vascular mechanisms.

We also found that greater occipital cortex volume at baseline was associated with slower decline in GDS. There is prior evidence of reduced gray matter volumes^40^, and functional connectivity and activation^41^ in the occipital lobe among those with depression. Loss of connectivity may be due in part to disruptions to the super longitudinal fasciculus, which connects the frontal, parietal, occipital, and temporal lobes^42^. However, the role of the occipital lobe remains unclear given its involvement in multiple processes, requiring further study to understand how occipital lobe volumes impact depressive symptom trajectories.

While our other results were not significant, most estimates were in the hypothesized direction, consistent with the hypothesis that lower cortical volume and less white matter integrity was linked to faster increases in GDS. Notably, prior literature typically examined the opposite direction of our hypotheses, the association of GDS with neuroimaging. For example, in prior work^43^, we found that lower GDS scores were associated with greater hippocampal volume at baseline. In another cross-sectional study of depression severity and hippocampal volume change (mean age = 69.7 years; age range = 60–88 years), smaller hippocampal volumes were associated with higher depressive symptom scores^11^. In testing amyloid with GDS trajectories, we did not find differences by amyloid SUVR or in brain specific regions. This is at odds with a study of 154 participants (age range = 61–89) from the Harvard Aging Brain Study, that found that increasing amyloid accumulation in the isthmus cingulate, middle frontal, and medial orbitofrontal cortices were associated with increasing GDS scores over an average 8.6 year follow up^12^. Conversely, a recent meta-analysis of 24 studies (mean age range = 61–78 years) found no cross-sectional associations between depressive symptom and amyloid measured via cerebrospinal fluid, PET or plasma^44^. The discrepancies between study findings may be attributed to differences in study duration, study population age as well as racial and ethnic makeup, and other neuropathological changes that are prevalent among nonagenarians and centenarians^45^ that could impact depressive symptoms via vascular mechanisms^15^. Additionally, our LifeAfter90 cohort may be reflective of an exceptionally aged cohort, potentially with less evidence of poor brain health and depressive symptoms by virtue of selection bias^46^. Moreover, the average follow up of approximately 1 year in our study may not have been sufficient to detect longitudinal changes by hippocampal and other volumes.

This study has several limitations. First, there may be outcome misclassification bias due to self-report of depressive symptoms in the GDS. This bias may result in measurement error either away or towards the null, if participants under- or over-reported symptoms. Future work should also account for depression history and other cognitive measures, which we were not able to assess here. Second, we did not have repeated imaging data, and thus limited to testing associations of baseline imaging with GDS change. This data would help clarify if depressive symptom change coincides with brain change and allow for analyses that simultaneously assess both trajectories^11,12^. Third, we did not assess the impact of incident dementia, which is associated with poor brain health^47^ and depressive symptoms^35^. Our results were attenuated after adjustment for time-varying cognitive function in sensitivity analyses attempting to account for this limitation. This suggests overadjustment bias which is known to attenuate associations^48^, and that this association potentially operates through cognitive function, suggesting its role as a mediator if brain change precedes cognitive change^49^. Larger studies with extended follow-up are needed to assess this longitudinal relationship using a causal mediation framework. Fourth, while there are noted relations between vascular risk factors and brain health^9,50^, we were unable to account for factors such as hypertension, diabetes, and dyslipidemia that may contribute to the vascular depression hypothesis^15^. Lastly, given that participants needed to have survived to age 90, generalizability of our findings to those of younger age is limited.

Our study had multiple strengths. The study population included racially and ethnically diverse participants aged ≥ 90 years. We used repeated assessments of depressive symptoms measured at a high frequency of approximately 6-month intervals, spanning up to 3.87 years. These data allowed us to assess within-person change of GDS scores in relation to baseline brain health biomarkers. Our depressive symptom assessment was based on a validated questionnaire^28,29^ that has been used in studies of participants aged ≥ 90 years^30^. We used IPSW to account for selection into the imaging cohort results and results were similar after applying IPSWs to conditionally account for demographic differences between the full and imaging cohorts, such as higher education levels (see Tables 1 and 2). Results were also similar after excluding participants with GDS scores suggestive of clinically significant depressive symptoms at baseline (see Supplementary Table 3). Lastly, our neuroimaging-based measures of brain health were derived from validated processes^20–27^. This allowed us to examine multiple biomarkers for a broader examination of different dimensions of brain health.

In this study of 225 racially and ethnically diverse adults between the ages of 90 to 103, greater occipital cortex volume and lower FW were associated with a slower increase in GDS scores. Our results suggest that cortical volume and white matter integrity may impact subsequent trajectories of depressive symptoms among those with exceptional longevity. Given the expected growth of the global population aged ≥ 90 years to 147 million by the year 2060^3^, improved understanding of the neurological underpinnings of depressive symptoms can support higher quality healthspan among those with exceptional longevity.

## Supplementary Information

Below is the link to the electronic supplementary material.

Supplementary Material 1

## Data Availability

Data are available upon approved request at https://sites.google.com/g.ucla.edu/khandle-study-site/home.

## Abbreviations

CI: Confidence interval
CSF: Cerebrospinal fluid
FA: Fractional anisotropy
FW: Free water
GDS: Geriatric Depression Scale
ICV: Intracranial volume
IPSW: Inverse probability of selection weights
KPNC: Kaiser Permanente Northern California
MRI: Magnetic resonance imaging
PET: Positron emission tomography
SD: Standard deviation
SENAS: Spanish and English Neuropsychological Assessment Scale
STROBE: Strengthening the Reporting of Observational Studies in Epidemiology
SUVR: Standardized uptake value ratio
WMH: White matter hyperintensities
