# Supplementary material for: Free water, occipital volume and changes in depressive symptoms in the LifeAfter90 study
Source: Sci Rep. 2026 May 23;16:23582. doi: 10.1038/s41598-026-54542-9 (PMC13421447; doi:10.1038/s41598-026-54542-9)
Supplement: Supplementary file 1 — Supplementary Material 1 [file 41598_2026_54542_MOESM1_ESM.docx]

# **Supplementary Online Content**

**Supplementary Methods.** Methods for MRI and amyloid accumulation and acquisition and processing

**Supplementary Table 1.** Description of 15-item Geriatric Depression Scale (GDS) items

**Supplementary Table 2.** Longitudinal association of 1 SD increase in brain biomarkers with Geriatric Depression Scale scores adjusted for time-varying cognitive function

**Supplementary Table 3.** Associations of 1 SD increase in brain biomarkers with Geriatric Depression Scale (GDS) over time, after excluding at different cutpoints

**Supplementary Table 4.** Interaction tests of gender and race and ethnicity as effect measure modifiers in longitudinal associations of 1 SD increase in brain biomarkers with Geriatric Depression Scale scores

**Supplementary Figure 1.** Median and interquartile range (IQR) of Geriatric Depressive Scale (GDS) scores across all study visits

**Supplementary Figure 2.** Predicted Geriatric Depression Scale (GDS) scores, where greater scores suggest greater depressive symptom severity, by z-scored hippocampal volume and over time adjusted for age, gender, race and ethnicity, and education.

**Supplementary Methods. Methods for MRI and amyloid accumulation and acquisition and processing**

MRI acquisition and processing were performed using standardized protocols in LifeAfter90. Briefly, MRIs were acquired using 3T Siemens TrioTrim or Prisma Fit models. neuroimaging-based measures of brain health were Full details on MRI acquisition and processing are found in There were 3 sequences: 1) T1-weighted volumetric MP-RAGE (3DT1): repetition time (TR) = 2500 ms, echo time (TE) = 2.98 ms, inversion time (TI) = 1100 ms, 192 slices, field of view (FOV) = 256 mm, acquisition matrix =256 x 256, slice thickness = 1 mm; 2) fluid attenuated inversion recovery (FLAIR): TR = 8800 ms, TE = 500 ms, TI = 2360 ms, 96 slices, FOV = 256 mm, acquisition matrix = 256 x 256, slice thickness = 2 mm; and 3) multi-shell DTI: TR = 6000 ms, TE = 87 ms, 48 slices, FOV = 256 mm, acquisition matrix = 96 × 96, slice thickness = 2.7 mm with a 2.7 mm gap. Diffusion weighted images were created with 13 gradient directions with gradient diffusion sensitivity of b = 500 s/mm2, 21 gradient directions with b = 1000 s/mm2, 15 gradient directions with b = 2000 s/mm2, and 3 images with b = 0 s/mm2.

Total intracranial volume (ICV) segmentation and quantification were performed using a convolutional neural network method described in detail elsewhere 1. Regional gray matter volumes were calculated using nonlinear co-registration of images to the Desikan-Killiany-Tourville atlas 2–4. WMHs were segmented using a Bayesian approach estimated from FLAIR images 5. Hippocampal masks were computed using a multi-atlas hippocampal segmentation algorithm 3. Free water (FW) and white matter fractional anisotropy (FA) maps were calculated from DTI 6,7. Mean FA and mean FW were generated from single-shell data using a previously published method 5.

To assess amyloid accumulation, PET scans used amyloid-binding fluorine-18 radiotracer florebatapir (Avid Radiopharmaceuticals) and were processed using Alzheimer’s Disease Neuroimaging Initiative protocols that are found online (see https://adni.loni.usc.edu/methods/pet-analysis-method/pet-analysis/). Mean standardized uptake value ratio (SUVR) was calculated to quantify regional tracer uptake across voxels relative to the whole cerebellar gray matter reference region 8.

**References**

1. Fletcher E, DeCarli C, Fan AP, Knaack A. Convolutional Neural Net Learning Can Achieve Production-Level Brain Segmentation in Structural Magnetic Resonance Imaging. Front Neurosci. 2021;15:683426. doi:10.3389/fnins.2021.683426

2. Desikan RS, Ségonne F, Fischl B, et al. An automated labeling system for subdividing the human cerebral cortex on MRI scans into gyral based regions of interest. NeuroImage. 2006;31(3):968-980. doi:10.1016/j.neuroimage.2006.01.021

3. Aljabar P, Heckemann RA, Hammers A, Hajnal JV, Rueckert D. Multi-atlas based segmentation of brain images: atlas selection and its effect on accuracy. Neuroimage. 2009;46(3):726-738. doi:10.1016/j.neuroimage.2009.02.018

4. Tustison NJ, Cook PA, Klein A, et al. Large-scale evaluation of ANTs and FreeSurfer cortical thickness measurements. Neuroimage. 2014;99:166-179. doi:10.1016/j.neuroimage.2014.05.044

5. Maillard P, Lu H, Arfanakis K, et al. Instrumental validation of free water, peak-width of skeletonized mean diffusivity, and white matter hyperintensities: MarkVCID neuroimaging kits. Alzheimers Dement (Amst). 2022;14(1):e12261. doi:10.1002/dad2.12261

6. Jenkinson M, Beckmann CF, Behrens TEJ, Woolrich MW, Smith SM. FSL. Neuroimage. 2012;62(2):782-790. doi:10.1016/j.neuroimage.2011.09.015

7. Hoy AR, Koay CG, Kecskemeti SR, Alexander AL. Optimization of a free water elimination two-compartment model for diffusion tensor imaging. Neuroimage. 2014;103:323-333. doi:10.1016/j.neuroimage.2014.09.053

8. Landau SM, Breault C, Joshi AD, et al. Amyloid-β imaging with Pittsburgh compound B and florbetapir: comparing radiotracers and quantification methods. J Nucl Med. 2013;54(1):70-77. doi:10.2967/jnumed.112.109009

| **Supplementary Table 1. Description of 15-item Geriatric Depression Scale (GDS) items** | |
| --- | --- |
| **Item** | **Question** |
| 1* | Are you basically satisfied with your life? |
| 2 | Have you dropped many of your activities and interests? |
| 3 | Do you feel that your life is empty? |
| 4 | Do you often get bored? |
| 5* | Are you in good spirits most of the time? |
| 6 | Are you afraid that something bad is going to happen to you? |
| 7* | Do you feel happy most of the time? |
| 8 | Do you often feel helpless? |
| 9 | Do you prefer to stay at home, rather than going out and doing new things? |
| 10 | Do you feel you have more problems with memory than most? |
| 11* | Do you think it is wonderful to be alive now? |
| 12 | Do you feel worthless the way you are now? |
| 13* | Do you feel full of energy? |
| 14 | Do you feel that your situation is hopeless? |
| 15 | Do you think that most people are better off than you are? |
| Note: * indicates a reverse scored item. GDS was derived from Yesavage et al^1^ and Sheikh and Yesavage.^2^  References for Supplementary Table1:   1. Yesavage JA, Brink TL, Rose TL, et al. Development and validation of a geriatric depression screening scale: a preliminary report. J Psychiatr Res. 1982;17(1):37-49. doi:10.1016/0022-3956(82)90033-4 2. Sheikh J, Yesavage J. Geriatric Depression Scale (GDS) Recent evidence and development of a shorter version. In: Brink T, ed. Clinical Gerontology : A Guide to Assessment and Intervention. Haworth Press; 1986:165-173. | |

| **Supplementary Table 2. Longitudinal association of 1 SD increase in brain biomarkers with Geriatric Depression Scale scores adjusted for time-varying cognitive function** | |
| --- | --- |
| **Brain biomarker by time** | **β (95% CI)** |
| Amyloid |  |
| SUVR | -0.14 (-0.37, 0.08) |
| Cerebrospinal fluid volume |  |
| Lateral ventricle | 0.15 (-0.07, 0.38) |
| Third ventricle | 0.04 (-0.19, 0.27) |
| Cortex volume |  |
| Frontal | 0.05 (-0.17, 0.27) |
| Occipital | -0.21 (-0.46, 0.03) |
| Parietal | -0.20 (-0.45, 0.05) |
| Temporal | -0.13 (-0.37, 0.10) |
| Gray matter volume |  |
| Total cerebrum gray | -0.12 (-0.35, 0.11) |
| Total hippocampus | -0.14 (-0.36, 0.08) |
| Left hippocampus | -0.13 (-0.34, 0.08) |
| Right hippocampus | -0.12 (-0.34, 0.11) |
| White matter integrity |  |
| Free water | 0.28 (0.03, 0.54) |
| Fractional anisotropy | -0.26 (-0.58, 0.06) |
| Log of WMH | 0.17 (-0.11, 0.44) |
| Note: All brain biomarkers were z-standardized and were residual values that accounted for intracranial volume. GDS is scored such that higher score suggest greater depressive symptom severity. Models included the main effect of the brain biomarker, and adjusted for age, gender, race and ethnicity, and education. Abbreviations: SUVR = standardized uptake value ratio, WMH = white matter hyperintensities. | |

| **Supplementary Table 3. Associations of 1 SD increase in brain biomarkers with Geriatric Depression Scale (GDS) over time, after excluding at different cutpoints** | | |
| --- | --- | --- |
|  | **Excluding n = 60 with GDS≥4** | **Excluding n = 39 with GDS≥5** |
| **Brain biomarker by time** | **β (95% CI)** | **β (95% CI)** |
| Amyloid |  |  |
| SUVR | -0.11 (-0.37, 0.15) | -0.11 (-0.36, 0.14) |
| Cerebrospinal fluid volume |  |  |
| Lateral ventricle | 0.20 (-0.07, 0.46) | 0.17 (-0.08, 0.41) |
| Third ventricle | 0.05 (-0.21, 0.32) | 0.03 (-0.22, 0.28) |
| Cortex volume |  |  |
| Frontal | 0.14 (-0.12, 0.40) | 0.15 (-0.09, 0.38) |
| Occipital | -0.27 (-0.54, -0.00) | -0.25 (-0.51, 0.01) |
| Parietal | -0.19 (-0.46, 0.09) | -0.14 (-0.41, 0.13) |
| Temporal | -0.04 (-0.30, 0.22) | -0.06 (-0.30, 0.19) |
| Gray matter volume |  |  |
| Total cerebrum gray | -0.09 (-0.35, 0.17) | -0.09 (-0.34, 0.16) |
| Total hippocampus | -0.13 (-0.39, 0.12) | -0.12 (-0.36, 0.11) |
| Left hippocampus | -0.12 (-0.38, 0.13) | -0.12 (-0.35, 0.11) |
| Right hippocampus | -0.12 (-0.37, 0.13) | -0.11 (-0.34, 0.13) |
| White matter integrity |  |  |
| Free water | 0.36 (0.06, 0.65) | 0.33 (0.04, 0.62) |
| Fractional anisotropy | -0.36 (-0.75, 0.02) | -0.29 (-0.66, 0.08) |
| Log of WMH | 0.23 (-0.09, 0.54) | 0.22 (-0.09, 0.53) |
| Note: All brain biomarkers were z-standardized and were residual values that accounted for intracranial volume. GDS is scored such that higher score suggest greater depressive symptom severity. Models included the main effect of the brain biomarker, and adjusted for age, gender, race and ethnicity, education, and time-varying executive function and verbal episodic memory, and a Visit 1 LifeAfter90 indicator to account for cognitive function testing practice effects. Abbreviations: SUVR = standardized uptake value ratio, WMH = white matter hyperintensities. | | |

| **Supplementary Table 4. Interaction tests of gender and race and ethnicity as effect measure modifiers in longitudinal associations of 1 SD increase in brain biomarkers with Geriatric Depression Scale scores** | | |
| --- | --- | --- |
| **Brain biomarker** | **P-value for interaction for gender** | **P-value for interaction for race and ethnicity** |
| Amyloid |  |  |
| SUVR | 0.98 | 0.40 |
| Cerebrospinal fluid volume |  |  |
| Lateral ventricle | 0.25 | 0.16 |
| Third ventricle | 0.55 | 0.06 |
| Cortex volume |  |  |
| Frontal | 0.59 | 0.82 |
| Occipital | 0.59 | 0.05 |
| Parietal | 0.15 | 0.84 |
| Temporal | 0.15 | 0.64 |
| Gray matter volume |  |  |
| Total cerebrum gray | 0.33 | 0.86 |
| Total hippocampus | 0.60 | 0.15 |
| Left hippocampus | 0.26 | 0.23 |
| Right hippocampus | 0.74 | 0.08 |
| White matter integrity |  |  |
| Free water | 0.62 | 0.69 |
| Fractional anisotropy | 0.45 | 0.51 |
| Log of WMH | 0.49 | 0.78 |
| Note: All brain biomarkers were z-standardized and were residual values that accounted for intracranial volume. GDS is scored such that higher score suggest greater depressive symptom severity. P-values for interaction were derived from testing models with and without a brain biomarker-by-modifier-by-time interaction using likelihood ratio tests. Models included the main effect of the brain biomarker, and adjusted for age, gender, race and ethnicity, and education, and includes inverse probability of selection weights. Abbreviations: SUVR = standardized uptake value ratio, WMH = white matter hyperintensities. | | |


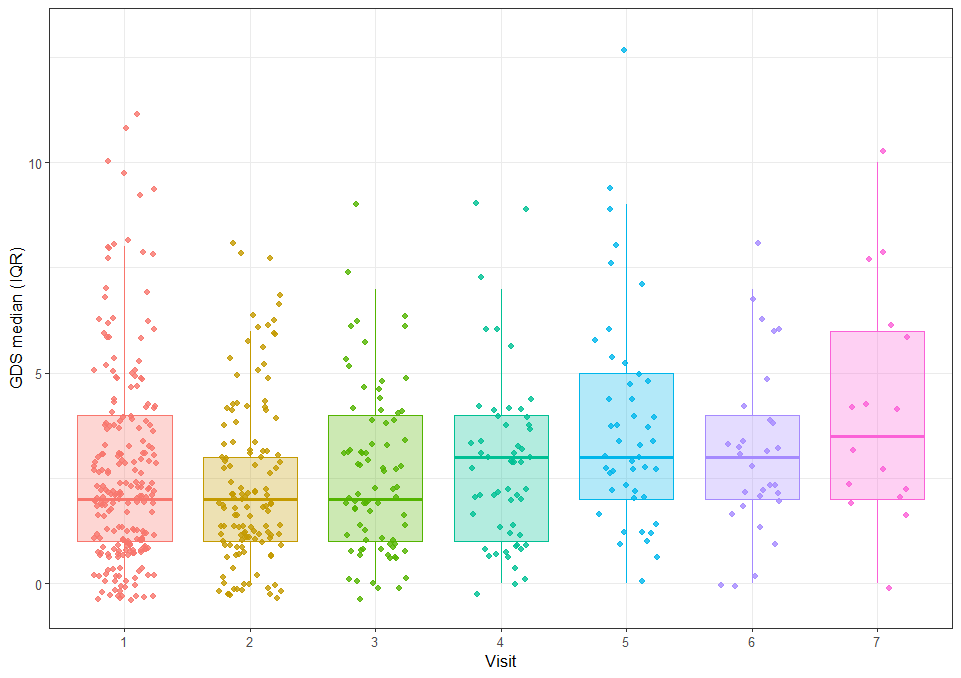


**Supplementary Figure 1. Median and interquartile range (IQR) of Geriatric Depressive Scale (GDS) scores across all study visits**

**
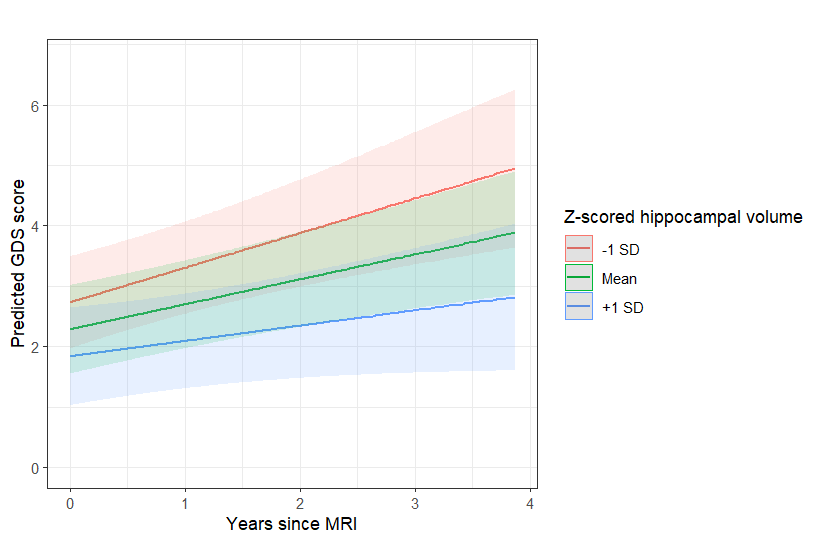
**

**Supplementary Figure 2. Predicted Geriatric Depression Scale (GDS) scores, where greater scores suggest greater depressive symptom severity, by z-scored hippocampal volume and over time adjusted for age, gender, race and ethnicity, and education.**
